# Young Myeloma Patients: A Systematic Review of Manifestations and Outcomes

**DOI:** 10.3390/curroncol30060396

**Published:** 2023-05-23

**Authors:** Mégane Tanguay, Christophe Dagenais, Richard LeBlanc, Imran Ahmad, Jean-Sébastien Claveau, Jean Roy

**Affiliations:** 1 Institut Universitaire d’Hémato-Oncologie et de Thérapie Cellulaire, Hôpital Maisonneuve-Rosemont, 5415 de l’Assomption, Montreal, QC H1T 2M4, Canada; megane.tanguay@umontreal.ca (M.T.); richard.leblanc.1@umontreal.ca (R.L.); imran.ahmad.1@umontreal.ca (I.A.); jean-sebastien.claveau@umontreal.ca (J.-S.C.); 2 Departement of Medicine, Université de Montréal, 2900 Édouard-Montpetit, Montreal, QC H3T 1J4, Canada; christophe.dagenais.med@ssss.gouv.qc.ca; 3 Service de Médecine Interne, Hôpital Notre-Dame, 1560 Sherbrooke Est, Montreal, QC H2L 4M1, Canada

**Keywords:** multiple myeloma, young patients, biology, outcome

## Abstract

Multiple myeloma usually affects older adults. However, younger patients constitute a significant subset as approximately 10% of cases occur in subjects younger than 50 years old. Young patients, who are underrepresented in the literature, are diagnosed during their most productive years of life, urging the need for tailored treatment approaches. This literature review aims to report recent studies specifically addressing young patients with a focus on characteristics at diagnosis, cytogenetics, treatments, and outcomes. We searched PubMed for studies involving young patients with multiple myeloma ≤50 years old. The time span of our literature review search was from 1 January 2010 to 31 December 2022. Overall, 16 retrospective studies were analyzed for this review. Young patients with multiple myeloma tend to have less advanced disease, more frequent light chain subtypes, and survive longer compared to their older counterparts. However, available studies included a limited number of patients; the newest revised international staging system was not used to stratify patients, cytogenetics varied from one cohort to another, and most patients did not receive contemporary triplet/quadruplet treatments. This review emphasizes the need to perform contemporary, large-scale retrospective studies to improve knowledge regarding the presentation and outcomes of young myeloma patients in the era of modern treatments.

## 1. Introduction

Despite several therapeutic advances in recent years, multiple myeloma (MM) remains a mostly incurable disease. The aims of current treatments are to relieve symptoms, reverse or avoid organ damage, improve quality of life, and prolong survival. The disease specifically affects older adults, with a median age at diagnosis of about 70 years [1,2]. Nevertheless, younger patients constitute a significant subset as approximately 10% of all cases occur in subjects younger than 50 years old [3]. The average years of life lost per patient is as high as 36 years for patients under 40 years and reaches up to 27 years for patients between the ages of 40 to 49 years. In contrast, the entire myeloma population loses 16.8 years of life on average to the disease [4]. In addition to a significantly shorter life expectancy, young patients are affected in their most productive years professionally and suffer a significant deterioration in their quality of life due to the disease [5]. 

Young myeloma patients are underrepresented in the literature, with small numbers buried in cohorts of older patients in clinical trials testing novel therapies. Few studies have specifically addressed their clinical characteristics and outcomes in recent years. Most are case reports or small cohorts spanning over several years, with only a minority of studies including a significant number of patients treated with contemporary regimens. Young patients with MM tend to better tolerate treatments with less therapy discontinuation or dose reductions, emphasizing that this unique subpopulation requires a tailored approach to treatment [6]. We believe there is a need to further understand the disease biology and optimal treatments in this younger subgroup. This literature review aims to report available studies specifically addressing young myeloma patients and underline the needs and shortcomings surrounding them with a focus on four aspects: patients’ characteristics at diagnosis, cytogenetics, treatments administered, and outcomes.

## 2. Methods

**Figure 1:**
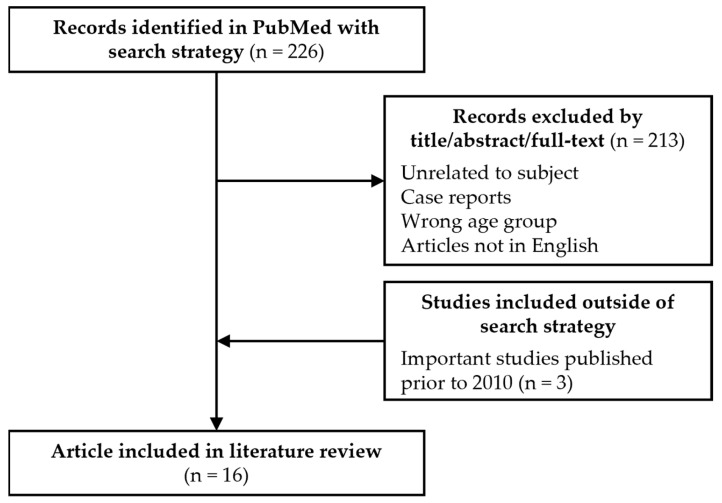
Consort diagram.

**Figure 2:**
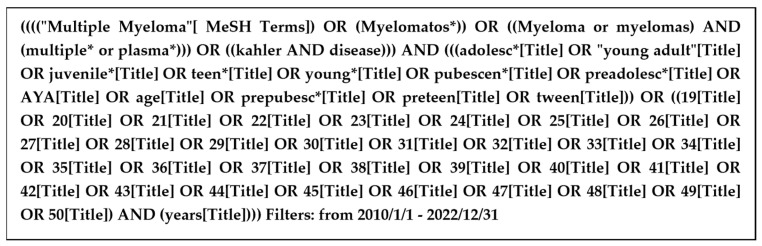
PubMed search strategy. * Truncation to find variations of a word.

We searched PubMed for studies involving MM in young patients (see Figure 1 and Figure 2 for search strategies). 

Owing to the lack of consensus on the definition of young patients, we elected, for the purpose of this review, to define them as ≤50 years old. The time span of our literature review search was from 1 January 2010 to 31 December 2022. We restricted the search to the last 12 years to include patients treated with novel therapies and risk stratified using the newest prognostic scores. PubMed was used to retrieve studies involving patients ≤50 years old. As shown in Figure 1, 226 studies were initially identified. Studies unrelated to MM, case reports, studies including only patients over 50 years, and studies in languages other than English were excluded.

The search strategy was developed by 2 reviewers (CD and MT), who screened the title and the abstract while creating a shortlist of studies for further evaluation. If a decision on inclusion was not possible on this basis, we obtained the full text of the article to assess eligibility. Disagreement in study selection was resolved through mutual discussion and consensus with a third author (JR). To increase the data concerning patient characteristics, three articles that included large cohorts of young patients were added to the analysis even if they were published prior to 2010 [7,8,9]. Overall, 16 retrospective studies were analyzed in this review.

Studies were classified into two groups based on the presence or absence of an older age group comparator. Different tables were created to collect relevant information: patients’ characteristics, cytogenetics, treatments, and outcomes. The patients’ characteristics from all 16 studies are available in Table 1 and Table 2. Non-secretory myeloma patients were not included in our analysis because they were rarely reported. Cytogenetic data were available in 11 studies (Table 3). More modern publications were included in the treatments and outcomes to focus on young patients treated with contemporary regimens. Only articles disclosing the 5-year overall survival (OS) or progression-free survival (PFS) were included to better highlight the long-term impact of MM in young patients, therefore 8 studies were retained in Table 4. Data on consolidation and maintenance were not consistently reported and therefore were not included.

## 3. Results

### 3.1. Disease Characteristics at Diagnosis

Characteristics at initial diagnosis including patients’ age, International Staging System (ISS), disease features, M protein isotype, and cytogenetics are presented in Table 1, Table 2 and Table 3. Overall, disease characteristics at diagnosis in the different studies were quite similar in terms of clinical and laboratory data. However, two factors seem to stand out in young patients: the light chain MM and ISS 1 subgroup.

While the incidence of light chain disease is approximately 15% in the general myeloma population [23], studies including only young patients have reported incidences ranging from 19 to 45% in patients ≤ 50 years [7,10,13,14,15,16,17], with only one study with a lower incidence of 11% [12]. Similarly, all studies comparing young and older patients have shown a higher proportion of light chain MM, including Corso et al. (<50 years: 12% vs. ≥50 years: 8%), Ludwig et al. (<50 years: 13% vs. ≥50 years: 10%, *p* = 0.002), Lu et al. (<50 years: 33% vs. ≥50 years 26%, *p* = 0.057), Dhakal et al. (≤50 years: 30% vs. >70 years: 18%), Nakaya et al. (<40 years: 33% vs. all patients: 16%, *p* = 0.021), and Pydi et al. (<40 years: 41% vs. all patients: 33%) [8,9,18,20,21,22]. 

Approximately 25% of all MMs are in the ISS 1 subgroup [16,24]. In some studies without an older age group comparator included in this review, a higher proportion of ISS 1 was reported, ranging from 32 to 68% [10,11,13,14,15,16,17]. One study reported a lower ISS 1 incidence of 13% [12]. Similarly, among studies comparing age groups, three reported a higher proportion of ISS 1 in younger patients including Ludwig et al. (<50 years: 36% vs. ≥50 years: 26%, *p* < 0.001), Lu et al. (<50 years: 30% vs. ≥50 years: 17%, *p* < 0.001), and Nakaya et al. (<40 years: 43% vs. all patients: 23%, *p* = 0.019) [9,18,21]. 

It remains unclear whether young patients diagnosed with MM have an increased incidence of a familial history of MM or secondary hematological malignancies. In Altieri et al.’s database study, the age at diagnosis of familial cases was lower compared to sporadic cases [25]. As per Vachon et al., there was an increased risk of MGUS (monoclonal gammopathy of undetermined significance) in first-degree relatives of probands with MGUS and MM. This increased prevalence was seen across all ages of relatives ≥ 40 years old [26]. In Clay-Gilmour et al.’s study, the familial risk of MGUS remained consistent regardless of the age at which the proband was diagnosed [27]. 

### 3.2. Cytogenetics

Table 3 highlights the different cytogenetic abnormalities of 11 recent studies included in this review. There is great heterogeneity in abnormalities tested and reported. For instance, Duek et al. reported t(11;14) in up to 68% of their young MM cohort (<50 years old) [15], which is much higher than the expected 15–20% found in the MM population [3,28]. As shown in Caulier et al.’s cohort, del (17p) and/or t(4;14) were present in 18% of their young patients (<40) and were a predictor of poor OS [16]. Jurczyszyn et al. found that patients aged 21–40 years had a higher prevalence of high-risk cytogenetics (del (17p) and t(4;14)) compared to patients 41–60 years old (32% vs. 17%, *p* = 0.007) [19]. Nakaya et al. also found a higher frequency of del (17p) in patients < 40 compared to the entire myeloma cohort (33% vs. 14% *p* = 0.008) [21]. In contrast, studies by Lu and Ludwig have observed similar frequencies of different clonal cytogenetic abnormalities [9,18]. 

### 3.3. Treatments and Outcomes

Treatments and outcomes are shown in Table 4. Patients included were treated over a span of several years, drugs used for induction were reported inconsistently, and older regimens were of common occurrence. Studies reporting induction treatments with a combination of a proteasome inhibitor (PI) and an immunomodulatory drug (IMID) ranged from 15 to 69% in young patients [10,11,13,14,16,17]. Autologous stem cell transplant (ASCT), currently considered a standard of care in young patients, was performed heterogeneously. Allogeneic SCT was seldom used. Median OS, reported in 4 studies, ranged from 61 to 175 months [10,13,16,17]. In studies with a comparator, all studies reported a longer 5-year OS in young patients [14,19,21].

## 4. Discussion

Whether MM of the young is a different disease entity remains unclear and a matter of debate. Different thresholds used to define disease characteristics at diagnosis such as creatinine, albumin, LDH, calcium level, number of lytic bone lesions, and cytogenetic abnormalities make comparisons between cohorts challenging. Nevertheless, it seems that young patients with MM tend to have less advanced disease (ISS 1) and present more frequently with the light chain subtype.

This review gathered the most recent studies available. However, only the older ISS classification was used. Now that cytogenetics are widely available, it would be important to focus on the revised-ISS (R-ISS) in young patients, which combines high LDH and B2-microglobulin, low albumin, and the presence of high-risk cytogenetic abnormalities including del (17p), t(4;14), and t(14;16) [29].

Cytogenetic risk stratification in MM is a powerful outcome predictor [30]. Primary genetic events, present in almost all myeloma cells at diagnosis, are divided into hyperdiploid and non-hyperdiploid rearrangements. Hyperdiploidy is characterized by trisomies of odd-numbered chromosomes (except for 1, 13, and 21) and it is usually associated with a more indolent disease course. The non-hyperdiploid changes involve translocations of the immunoglobulin heavy-chain (IGH) gene locus on chromosome 14 with several different partner genes, usually leading to poorer prognosis, although there is heterogeneity depending on the translocation partner gene [31] Secondary chromosomal aberrations, typically occurring in subclones, develop as the disease progresses. Copy number abnormalities and gene mutations such as deletion of TP53 represent secondary changes leading to a more aggressive disease course [32,33]. MM is therefore a multistep process starting from MGUS, to smoldering myeloma, then evolving into MM as genetic events accumulate. The specific chromosomal aberrations in young patients are crucial since they can reflect tumor evolution and response to certain therapies. Interphase fluorescence in situ hybridization (iFISH) is the technique of choice for cytogenetic analysis. Besides del (17p) deletion, t(4;14), and t(14;16), young patients should also be screened for other cytogenetic abnormalities associated with high-risk disease, such as t(14;20) and chromosome 1 abnormalities [29,32].

At the present time, it remains unclear whether young myeloma patients are more prone to have high-risk cytogenetics for several reasons. First, the number of patients included was limited. The techniques differed from one cohort to the other, while older studies resorted to conventional cytogenetics and more contemporary studies used iFISH. Importantly, the details of methods used were not universally reported, including plasma cell purification and iFISH positivity cut-offs. Finally, the MM panels used in each study were usually not described and the presence or absence of multiple cytogenetic abnormalities (double or triple hit defining a very high-risk group) was often not clearly specified [34,35,36]. Further modern, large-scale studies are necessary to understand the genetic landscape of young patients with MM. More advanced techniques such as next-generation sequencing (NGS) should also aid in our understanding.

Myeloma survival has improved in the past decade [37]. In young patients eligible for transplantation, a combination of a PI, an IMID, and dexamethasone, such as the bortezomib, lenalidomide, and dexamethasone (VRd) triplet should be used [38,39,40,41]. Quadruplet therapy, with the addition of daratumumab (D), a monoclonal antibody targeting CD38, has shown promising results in different clinical trials [42,43,44] and is currently used by some centers in patients with high-risk MM [45]. For transplant ineligible patients, initial treatments with combinations of VRd and DRd followed by maintenance are standards of care [46,47].

The outcome of young MM patients is difficult to predict for several reasons. Only a small subset has received contemporary regimens, emphasizing the urgent need for modern studies. ASCT was also heterogeneously used, possibly due to resource limitations in some centers. It is therefore impossible to show prognosis differences between patients who have received ASCT and those who have not. Allogeneic SCT was a rare occurrence. Considering that it is the only potential curative treatment approach with improved nonrelapse mortality over time, it could be an interesting strategy for these patients [48,49]. Long-term survival data are lacking in most studies, which is of paramount importance in young patients at risk of developing long-term complications such as secondary malignancies or infections due to prolonged immunosuppression. The role of minimal residual disease, which could be a better predictor of longer PFS and potentially OS, remains unclear in young patients [50,51].

The OS of younger patients has been shown to be longer in most studies, possibly due to a higher incidence of ISS 1, better tolerance to more intensive therapies, less frequent preexistent comorbidities, and reporting bias. Nevertheless, young patients have a near 70-fold increase in mortality compared to the general population [16]. Other studies did not find differences in survival, possibly due to different induction treatments, less frequent use of first line transplants, or higher prevalence of patients with high-risk cytogenetics [10,20].

## 5. Conclusions and Future Directions

There is evidence that young patients with MM tend to have less advanced disease (ISS 1), more frequent light chain subtype, and survive longer compared to their older counterparts. It remains unclear whether their disease characteristics at presentation are distinct, due to unknown R-ISS distribution, incomplete cytogenetic comparisons with older patients, and limited information on the impact of current standard treatments. This review emphasizes the need to perform contemporary, large-scale retrospective studies to improve knowledge regarding their presentation and outcomes in the era of modern treatments. Young patients should be enrolled in clinical trials and specific stratification of different age groups should be conducted to further understand the characteristics and outcomes of their disease. These patients are diagnosed during their most productive years of life and suffer from significantly higher personal, familial, professional, and economic burdens compared to older patients, urging the need for tailored treatment approaches. Survivorship of young patients with MM deserves particular attention given their long disease journey with multiple potential complications, including secondary malignancies. Further studies will elucidate if innovative cellular therapies such as upfront CAR-T cells or other novel cellular therapies could be beneficial in this population.

## Figures and Tables

**Table 1:** Patients’ characteristics at diagnosis included in studies without a comparator.

	Bladé 1996 [7]	Shin 2017 [10]	Ravi 2018 [11]	Yanamandra 2018 [12]	Jurczyszyn 2019 [13]	Pál 2020 [14]	Duek 2021 [15]	Caulier 2021 [16]	Bao 2022 [17]
*n*	72	32	212	40	52	16	23	214	258
Country	USA	South Korea	USA	India	Europe, USA, Brazil, Hong Kong	Hungary	Israel	France, Belgium	USA
Years of diagnosis	1956–1992	2000–2015	2005–2015	2010–2015	1989–2016	2006–2015	2009–2014	2000–2015	1992–2019
Patients’ age (years)	<40	≤40	≤50	<40	≤30	≤40	<50	≤40	<50
Median age (range)	36 (19–39)	37 (17–40)	45 (22–49)	38 (18–39)	28 (8–30)	39 (31–40)	41.5 (27–49)	37.2 (18.6–40.9)	46 (17–50)
Male/female	50/22	19/13	129/83	26/14	35/17	10/6	17/6	137/77	165/93
ISS									
I	NA	10/31 (32)	74/212 (35) ^b^	5/40 (13)	32/47 (68)	7/16 (44)	5/14 (36)	99/189 (52)	89/212 (42)
II	NA	15/31 (48)	46/212 (22) ^b^	7/40 (18)	7/47 (15)	5/16 (31)	6/14 (43)	52/189 (28)	66/212 (31)
III	NA	6/31 (19)	48/212 (23) ^b^	28/40 (70)	8/47 (17)	4/16 (25)	3/14 (21)	38/189 (20)	57/212 (27)
Disease features at diagnosis									
Anemia (<100 g/L)	NA	9/31 (29)	NA	21/40 (53)	13/43 (30)	2/16 (13)	6/18 (33)	71/202 (35)	NA
Kidney disease ^a^	15/52 (29)	4/32 (13)	NA	12/40 (30)	4/22 (18)	2/16 (13)	3/18 (17)	34/200 (17)	NA
Low albumin ^a^	8/49 (16)	9/32 (28)	NA	NA	11/41 (27)	NA	8/18 (44)	NA	NA
Hypercalcemia ^a^	16/53 (30)	9/32 (28)	NA	9/37 (24)	6/42 (14)	3/16 (19)	1/18 (6)	25/195 (13)	NA
Lytic bone lesions	44/65 (68)	27/31 (87)	NA	16/37 (59)	36/44 (82)	14/16 (88)	16/18 (89)	149/200 (75)	NA
Elevated ß2MG ^a^	18/33 (55)	14/29 (48)	NA	NA	11/41 (27)	NA	NA	NA	NA
Protein isotype									
Heavy chain			NA						
IgG	34/66 (51)	14/30 (47)	NA	(76)	27/49 (55)	8/16 (50)	11/22 (50)	130/162 (80)	121/258 (47)
IgA	7/66 (11)	5/30 (17)	NA	(11)	9/49 (18)	3/16 (19)	2/22 (9)	28/162 (17)	53/258 (21)
IgD	4/66 (6)	2/30 (7)	NA	NA	NA	0/16 (0)	NA	3/162 (2)	NA
IgM	NA	NA	NA	(3)	NA	0/16 (0)	NA	1/162 (0.6)	NA
Light chain only	21/66 (32)	9/30 (30)	NA	(11)	11/49 (22)	3/16 (19)	10/22 (45) ^c^	51/213 (24)	72/258 (28)

^a^ Variable cut-off; ^b^ Numbers not adding to 100% due to missing data; ^c^ One case with isotypes IgG kappa and free lambda; Abbreviations: ß2MG: Beta-2 microglobulin; ISS: International Staging System; NA: not available. Numbers in parentheses represent percentages.

**Table 2:** Patients’ characteristics at diagnosis included in studies with a comparator.

	Corso 1998 [8]		Ludwig 2008 [9]		Lu 2016 [18]		Jurczyszyn 2016 [19]		Dhakal 2017 [20]		Nakaya 2020 [21]		Pydi 2021 [22]	
*n*	356		10,549		940		1089		191		2303		280	
Country	Italy		North America, Europe, Japan		China		Europe, USA		USA		Japan		India	
Years of diagnosis	1973–1994		1981–2002		2008–2011		2000–2015		2000 to 2015		1998–2018		2013–2018	
Age studied (years)	<50	≥50	<50	≥50	<50	≥50	21–40	41–60	≤50	>70	<40	All	≤40	All patients
n (by age group)	61	295	1689	8860	194	746	173	916	86	105	26	2277	22	258
Median age (range)	45 (33–49)	63 (50–87)	36 (20–49)	62 (50–93)	46 (20–49)	62 (50–88)	37 (21–40)	55 (41–60)	46 (32–50)	73 (71–79)	36 (20–39)	74 (20–96)	33.5 (18–40)	56 (18–84)
Male/female	32/29	169/126	1023/666	5014/3846	113/81	457/289	104/69	510/406	70/16	58/47	13/13	1116/1161	14:8	NA
ISS														
I	NA	NA	492/1267 (39)	1790/6776 (26) *p* < 0.001	(30)	(17) *p* < 0.001	71/151 (47)	303/729 (42) *p* = 0.40	15/86 (17) ^d^	29/105 (27) *p* = 0.31 ^d^	(43)	(23) *p* = 0.019	(18)	(17)
II	NA	NA	438/1267 (35)	2675/6776 (39) *p* < 0.001	(31)	(32) *p* = 0.774	50/151 (33)	280/729 (38)	22/86 (26) ^d^	22/105 (21) ^d^	(38)	(40) *p* = 0.910	(32)	(33)
III	NA	NA	337/1267 (27)	2311/6776 (34) *p* < 0.001	(39)	(51) *p* 0.007	30/151 (20)	149/729 (20)	20/86 (23) ^d^	28/105 (27) ^d^	(19)	(37) *p* = 0.022	(50)	(50)
Disease features at diagnosis														
Anemia (<100 g/L)	NA	NA	596/1614 (37)	3465/8539 (41) *p* = 0.006	(56)	(61) *p* = 0.265	53/173 (31)	247/925 (27) *p* = 0.29	NA	NA	6/26 (23)	NA	(68)	(63)
Kidney disease ^a^	5/61 (8)	41/295 (14)	240/1594 (15)	1484/8573 (17) *p* = 0.028	(21)	(25) *p* = 0.300	40/160 (25)	265/855 (31) *p* = 0.13	NA	NA	11/26 (42)	NA	(50)	(36)
Low albumin ^a^	NA	NA	458/1396 (33)	3276/7912 (41) *p* < 0.001	(37)	(58) *p* < 0.001	NA	NA	NA	NA	NA	NA	NA	NA
Hypercalcemia ^a^	4/61 (6)	17/295 (6)	481/1445 (33)	2652/7870 (34) *p* = 0.762	NA	NA	26/160 (16)	86/668 (13) *p* = 0.26	NA	NA	1/26 (4)	NA	(9)	(26)
Lytic bone lesions	26/61 (43) ^b^	100/295 (34) ^b^	617/1292 (48) ^c^	3457/7423 (47) ^c^ *p* = 0.431	82/109 (75) ^c^	334/403 (83) *p* = 0.569 ^c^	139/170 (82)	644/868 (74) *p* = 0.04	NA	NA	18/26 (69)	NA	(59)	(76)
Elevated ß2MG ^a^	NA	NA	613/1377 (45)	4141/7061 (59) *p* < 0.001	(46)	(62) *p* < 0.001	NA	NA	NA	NA	NA	NA	NA	NA
Protein isotype														
Heavy chain														
IgG	40/61 (65)	197/295 (67)	924/1538 (60)	4853/8091 (60) *p* = 0.943	75/194 (39)	341/746 (46) *p* = 0.078	107/156 (69)	375/632 (59) *p* = 0.10	34/86 (40)	56/105 (53) *p* = 0.06	(45)	(58) *p* = 0.237	(50)	(55)
IgA	11/61 (18)	59/295 (20)	318/1538 (21)	2009/8091 (25) *p* < 0.001	28/194 (14)	141/746 (19) *p* = 0.149	26/156 (17)	127/632 (20)	9/86 (10)	21/105 (20)	(11)	(22) *p* = 0.080	(5)	(12)
IgD	1/61 (1)	3/295 (1)	43/1538 (3)	251/8091 (3) *p* = 0.522	20/194 (10)	41/746 (5.5) *p* = 0.015	1/156 (0.6)	16/632 (3)	NA	NA	(4)	(1) *p* = 0.375	NA	NA
IgM	NA	NA	NA	NA	NA	NA	NA	NA	NA	NA	NA	NA	NA	NA
Light chain only	8/61 (12)	26/295 (8)	197/1538 (13)	824/8091 (10) *p* = 0.002	64/194 (33)	195/746 (26) *p* = 0.057	NA	NA	26/86 (30)	19/105 (18)	(33)	(16) *p* = 0.021	(41)	(33)

^a^ Variable cut-offs; ^b^ Extensive lytic lesions; ^c^ more than 3 bone lesions; ^d^ numbers not adding to 100% due to missing data; Abbreviations: ß2MG: Beta-2 microglobulin; ISS: International Staging System; NA: not available. Numbers in parentheses represent percentages.

**Table 3:** Cytogenetics at diagnosis.

	Studies without a Comparator							Studies with a Comparator							
	Shin 2017 [10]	Jurczyszyn 2019 [13]	Pál 2020 [14,15]	Duek 2021 [15]	Caulier 2021 [16]	Pydi 2021 [22]	Bao 2022 [17] ^h^	Ludwig 2008 [9]		Jurczyszyn 2016 [19]		Lu 2016 [18]		Nakaya 2020 [21]	
Age studied (years)	≤40	≤30	≤40	<50	≤40	≤40	<50	<50	≥50	21–40	41–60	<50	≥50	<40 years	All patients Median 74
Hyperdiploid	NA	NA	6/11 (55)	1/22 (4.5)	NA	NA	NA	NA	NA	NA	NA	3/33 (9)	8/120 (7)	NA	NA
Non-hyperdiploid	NA	19/21 (90)	NA	NA	NA	NA	Hypodiploid 5/210 (2)	NA	NA	NA	NA	4/33 (12)	24/120 (20)	NA	NA
t(11;14)	NA	1/20 (5)	NA	15/22 (68) ^a,b^	9/35 (26)	2/7 (29)	42/210 (20)	NA	NA	NA	NA	NA	NA	1/5 (20)	83/316 (26) *p* = 0.461
t(14;16)	0/11 (0)	NA	NA	0/22 (0)	1/39 (2.5)	1/7 (14)	NA	NA	NA	NA	NA	NA	NA	0/7 (0)	27/532 (5) *p* = 0.063
t(14;20)	0/6 (0)	NA	NA	0/22 (0)	NA	NA	NA	NA	NA	NA	NA	NA	NA	NA	NA
t(8;14)	NA	NA	NA	NA	NA	NA	4/210 (2)	NA	NA	NA	NA	NA	NA	NA	NA
t(4;14)	1/10 (10)	0/20 (0)	3/11 (27)	0/22 (0)	19/156 (12) ^e^	1/7 (14)	15/210 (7)	NA	NA	26/81 (32) ^j^	31/181 (17) ^j^ *p* = 0.007	NA	NA	2/8 (25)	168/802 (21) *p* = 0.659
del (17p)/17 delp53	1/9 (11)	2/21 (10)	2/11 (18)	1/22 (4.5)	17/141 (12) ^e^	1/7 (14)	15/210 (7)	NA	NA			17/91 (19) ^i^	61/351 (17) *p* 0.771 ^i^	3/9 (33)	86/606 (14) *p* = 0.008
+ or amp 1q21/1q gain	4/15 (27)	2/17 (12)	NA	NA	17/56 (30) ^f^	NA	48/210 (23)	NA	NA	NA	NA	49/87 (56) ^i^	139/313 (44) *p* = 0.064 ^i^	NA	NA
del (1p32)	NA	NA	NA	1/22 (4.5) ^c^	8/46 (17) ^g^	NA	NA	NA	NA	NA	NA	NA	NA	NA	NA
del (13q)/ del 13	4/17 (24)	8/26 (31)	NA	9/22 (40) ^d^	NA	3/7 (43)	72/210 (34)	32/53 (60) ^i^	150/320 (47) *p* = 0.069 ^i^	NA	NA	13/37 (35) ^i^	58/141 (41) *p* = 0.507 ^i^	4/8 (50)	211/435 (49) *p* = 1.000
								17/109 (16) ^ii^	45/345 (13) *p* = 0.499 ^ii^			3/33 (9) ^ii^	9/120 (8) *p* = 0.767 ^ii^		
del (9)	1/16 (6)	NA	NA	NA	NA	NA	NA	NA	NA	NA	NA	NA	NA	NA	NA

NA: not available. ^a^ sole aberration in 32%; ^b^ 3 also had IgH rearrangement, 6 also had del 13q, 1 also had delp53; ^c^ 1 also IgH rearranged; ^d^ 1 also had delp53, 6 also t(11;14), 3 also IgH rearranged, 1 also del 16q; ^e^ 2 patients had t(4;14) and del (17p); ^f^ Associated with high-risk cytogenetics in 5 patients; ^g^ Associated with high-risk cytogenetics in 2 patients and associated with +1q in 5 patients; ^h^ 7% had ≥2 high-risk chromosomal abnormalities; ^i^ by FISH; ^ii^ by conventional cytogenetics; ^j^ del (17p) and t(4;14) merged. Numbers in parentheses represent percentages.

**Table 4:** Treatments and outcomes.

	Studies without a Comparator					Studies with a Comparator					
	Shin 2017 [10]	Ravi 2018 [11]	Jurczyszyn 2019 [13]	Caulier 2021 [16]	Bao 2022 [17]	Jurczyszyn 2016 [19]		Nakaya 2020 [21]		Pál 2020 [14]	
*n*	32	212	52	214	258	173	916	26	2277	16	296
Age studied (years)	≤40	≤50	≤30	≤40	<50	21–40	41–60	<40	All	≤40	>40
Year of diagnosis	2000–2015	2005–2015	2006–2016	2000–2015	1992–2019	2000–2015		1998–2018		2006–2015	
Induction treatments (%)						Novel agents, unspecified					
PI based	10	45 ^b^	41	30	22	NA	NA	NA	NA	6	NA
IMID based	37	32 ^b^	24	1	10	NA	NA	NA	NA	13	NA
PI + IMIDs	40 ^a^	15 ^b^	21	37	27	NA	NA	NA	NA	69	NA
Other (chemotherapy, melphalan, dexamethasone only)	13	6 ^b^	15	26	41	NA	NA	NA	NA	13	NA
Radiotherapy only	NA	NA	NA	6	NA	NA	NA	NA	NA	NA	NA
Transplant											
ASCT (%) 1st line	62	NA	62	NA	NA	NA	NA	NA	NA	88	NA
ASCT (%)—at any stage	79	52	NA	93	87	11	89	39	NA	NA	NA
Allo-SCT (%)	0	NA	3	25	5 ^c^	NA	NA	42 ^d^	NA	NA	NA
Survival data											
Median follow-up (months)	64	69.6	86	76	93.6	51		78		NA	NA
Median OS (months)	61	NA	166	175	112.8	NA	NA	NA	NA	NA	NA
5-years OS (%)	54	70	77	84	86 (NHBP) 66 (NHWP)	83	67 *p* < 0.001	71	56	83	53
Median PFS (months)	16	NA	NA	41	38.4 (NHWP), 70.8 (NHPB)	NA	NA	NA	NA	NA	NA
5-years PFS (%)	14	28	NA	NA	NA	NA	NA	NA	NA	48	35

^a^ VAD +/− VTD; ^b^ numbers not adding to 100% due to missing data; ^c^ ASCT followed by Allo-SCT ^d^ 23% with a combination of ASCT and allo-SCT + 19% allo-SCT. ASCT: autologous stem cell transplant; Allo-SCT: allogeneic stem cell transplant; NHBP: non-hispanic black people; NHWP: non-hispanic white people; OS: overall survival; PFS: progression-free survival.
